# Supplementary material for: The interactome of intact mitochondria by cross-linking mass spectrometry provides evidence for coexisting respiratory supercomplexes
Source: Mol Cell Proteomics. 2017 Dec 8;17(2):216–32. doi: 10.1074/mcp.RA117.000470 (PMC5795388; doi:10.1074/mcp.RA117.000470)
Supplement: Supplemental Data [file supp_RA117.000470_133922_0_supp_23978_fzffwf.zip › spectra_annotation/mito_DR_spectra_annotation/63-1-3-1-1-1.pdf]

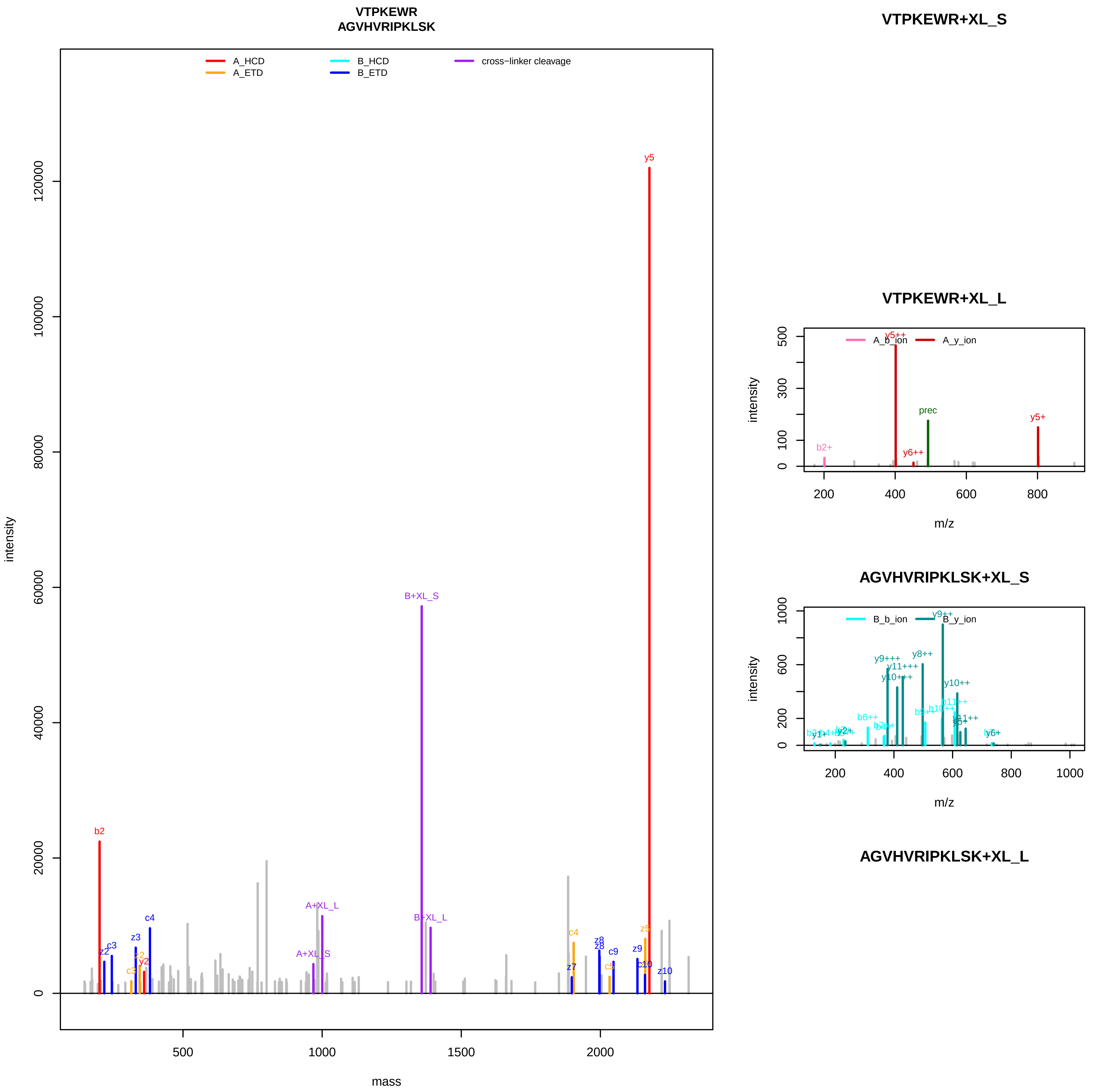

VTPKEWR
AGVHVRIPKLSK
A_HCD
A_ETD
B_HCD
B_ETD
cross−linker cleavage
y5
B+XL_S
b2
A+XL_L
B+XL_L
A+XL_S
c4
z3
c3
z2
c3
z2
y2
c4
z8
c9
z9
z5
c5
z7
c10
z10
intensity
mass
VTPKEWR+XL_S
VTPKEWR+XL_L
A_b_ion
A_y_ion
y5++
prec
y5+
y6++
b2+
m/z
AGVHVRIPKLSK+XL_S
B_b_ion
B_y_ion
y9++
y8++
y9+++
y11+++
y10+++
y10++
b11++
b10++
b6++
y11++
y5+
y6+
y2+
y1+
m/z
AGVHVRIPKLSK+XL_L
